# Supplementary material for: Enzymatic Hydrolysate of Cinnamon Waste Material as Feedstock for the Microbial Production of Carotenoids
Source: Int J Environ Res Public Health. 2021 Jan 28;18(3):1146. doi: 10.3390/ijerph18031146 (PMC7908450; doi:10.3390/ijerph18031146)
Supplement: Supplementary file 1 [file ijerph-18-01146-s001.zip › ijerph-1060076-supplementary.pptx]

## Slide 1
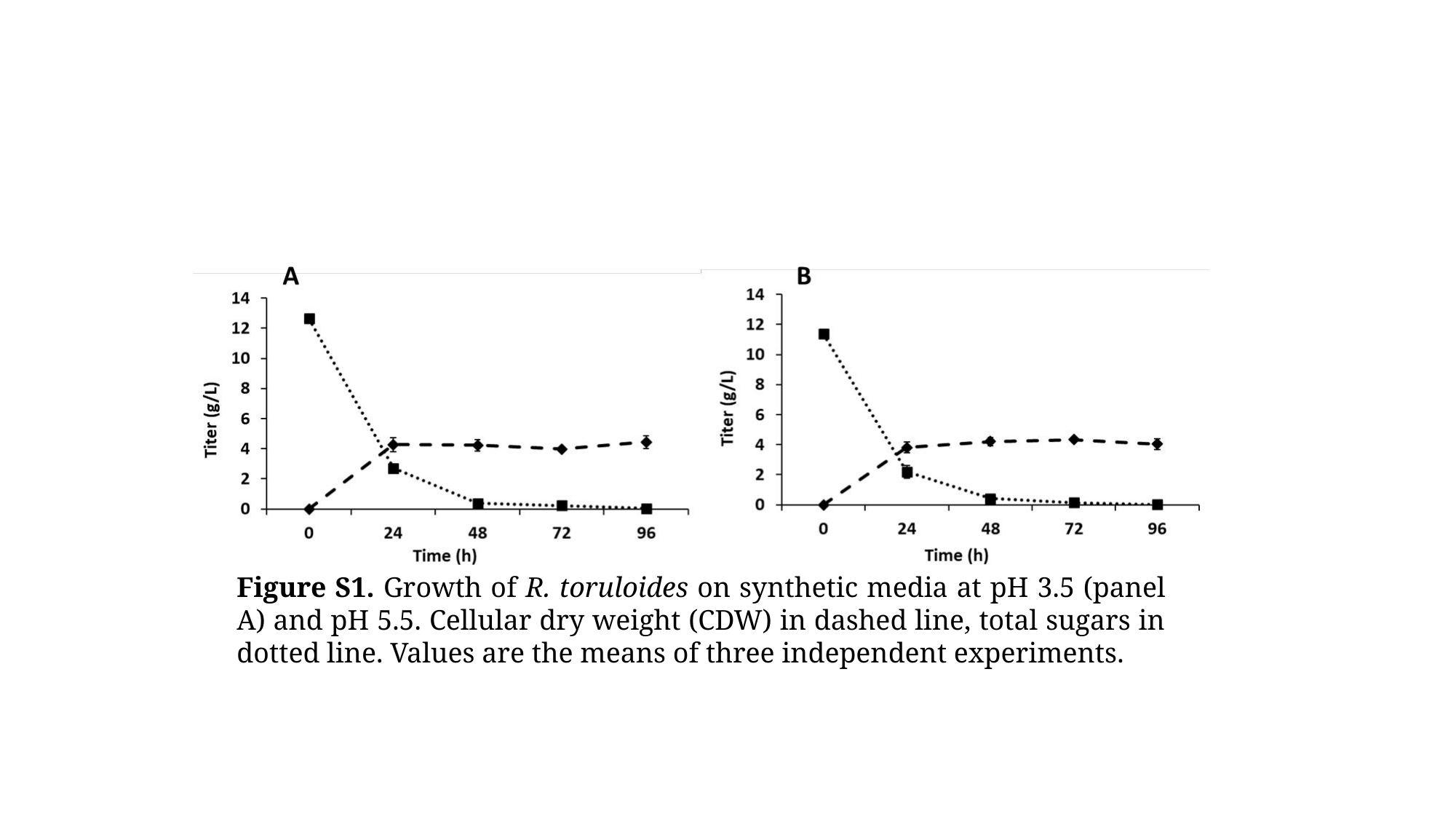

Figure S1. Growth of R. toruloides on synthetic media at pH 3.5 (panel A) and pH 5.5. Cellular dry weight (CDW) in dashed line, total sugars in dotted line. Values are the means of three independent experiments.

## Slide 2
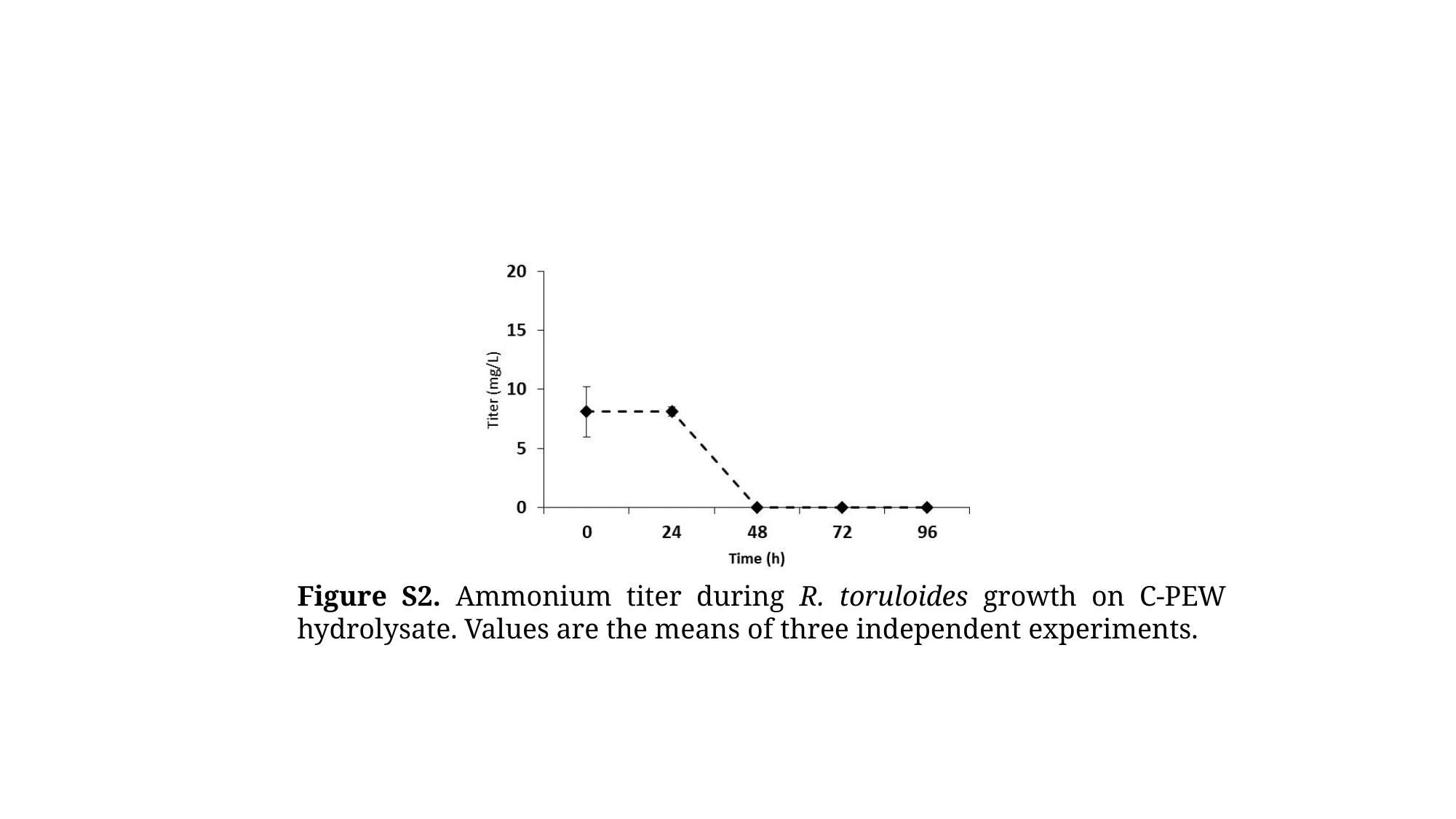

Figure S2. Ammonium titer during R. toruloides growth on C-PEW hydrolysate. Values are the means of three independent experiments.
